# COVID-19 Worsens Chronic Lumbosacral Radicular Pain—Case Series Report

**DOI:** 10.3390/ijerph19116410

**Published:** 2022-05-25

**Authors:** Róbert Illéš, Juraj Chochol, Andrej Džubera, Alica Chocholová, Erika Zemková

**Affiliations:** 1 Department of Neurosurgery, Slovak Medical University and University Hospital—St. Michael’s Hospital, Satinského 1, 811 08 Bratislava, Slovakia; robert.illes@nsmas.sk (R.I.); andrej.dzubera@nsmas.sk (A.D.); 2 Faculty of Medicine, Slovak Medical University in Bratislava, Limbová 12, 833 03 Bratislava, Slovakia; 3 Department of Paediatric Haematology and Oncology, National Institute of Children’s Diseases, Limbova 1, 833 40 Bratislava, Slovakia; alica.chocholova@nudch.eu; 4 Department of Biological and Medical Sciences, Faculty of Physical Education and Sport, Comenius University in Bratislava, Nábrežie Armádneho Generála Ludvíka Svobodu 9, 814 69 Bratislava, Slovakia; erika.zemkova@uniba.sk; 5 Sports Technology Institute, Faculty of Electrical Engineering and Information Technology, Slovak University of Technology, Ilkovičova 3, 812 19 Bratislava, Slovakia; 6 Faculty of Health Sciences, University of Ss. Cyril and Methodius in Trnava, Rázusova 14, 921 01 Trnava, Slovakia

**Keywords:** SARS-CoV-2, neurotropism, radicular irritation, neural roots irritation, radiculitis

## Abstract

The knowledge of the COVID-19 symptomatology has increased since the beginning of the SARS-CoV-2 pandemic. The symptoms of nervous system involvement have been observed across the spectrum of COVID-19 severity. Reports describing difficulties of nerve roots are rare; the affection of brain and spinal cord by SARS-CoV-2 is of leading interest. Our aim therefore is to describe the radicular pain deterioration in the group of nine chronic lumbosacral radicular syndrome sufferers in acute COVID-19. The intensity of radicular pain was evaluated by the Visual Analogue Scale (VAS). The VAS score in acute infection increased from 5.6 ± 1.1 to 8.0 ± 1.3 (Cohen’s *d* = 1.99) over the course of COVID-19, indicating dramatic aggravation of pain intensity. However, the VAS score decreased spontaneously to pre-infection levels after 4 weeks of COVID-19 recovery (5.8 ± 1.1). The acute SARS-CoV-2 infection worsened the pre-existing neural root irritation symptomatology, which may be ascribed to SARS-CoV-2 radiculitis of neural roots already compressed by the previous disc herniation. These findings based on clinical observations indicate that the neurotropism of novel coronavirus infection can play an important role in the neural root irritation symptomatology deterioration in patients with chronic pre-existing lumbosacral radicular syndrome.

## 1. Introduction

The COVID-19 pandemic spread from Wuhan (China) worldwide rapidly with 509 211 909 cases reported and led to 6 217 091 deaths worldwide (24 April 2022) [1]. From the beginning of SARS-CoV-2 pandemic, the knowledge about the COVID-19 symptomatology has been growing. The view of COVID-19 as a primary acute respiratory infection [2,3] has changed since the non-respiratory symptoms and long consequences were reported [4,5]. Nowadays, it is eminent that COVID-19 patients exhibit involvement and impairment in the structure and function of multiple organs, the nervous system included [4,6]. The heterogeneous neurological symptoms have been observed across the spectrum of COVID-19 severity. The brain [7], spinal cord [8,9,10,11], and peripheral nerves [7,12] can be affected in acute and chronic COVID-19 [4,5]. The SARS-CoV-2 associated back difficulties and radicular pain are of sparse occurrence in literature [13,14].

The neurological symptoms are present in more than 35% of SARS-CoV-2 infections, although determining the exact epidemiological data is difficult since the accurate reporting of neural symptoms is complicated [15]. Neurological impairment can be observed in acute [4,15,16,17] and as well as in long COVID-19 [5] and are of wide variability and severity. The most frequent central nervous system signs reported are headache, encephalitis, palsy, seizures, and impaired consciousness [15,16,17]. The olfactory nerve impairment leads the iconic feature of SARS-CoV-2 infection, anosmia and ageusia [18,19,20]. Moreover, the affection of spinal cord in COVID-19 can lead to several acute myelitis [8,9,10,11] and Guillain–Barré syndrome [12,21]. The paresthesia was seen [22] as peripheral nervous system involvement.

There is sparse information of neural root impairment in COVID-19. Only anecdotal clinical case reports of back pain in acute [13] and long COVID-19 [14] were presented. Back pain with radicular or pseudoradicular syndrome was the first complaint in acute SARS-CoV-2 infection. Back pain with radicular or pseudoradicular syndrome vanished after SARS-CoV-2 recovery in the majority of cases [13] spontaneously. On the other hand, the radicular pain can become chronic and neurosurgical intervention can be necessary [14].

Radicular pain is defined as “pain perceived as arising in a limb or the trunk wall caused by ectopic activation of nociceptive afferent fibers in a spinal nerve or its roots or other neuropathic mechanisms [23]”. In general, it can be said that radicular pain is caused by lesions that directly compromise the dorsal root ganglion mechanically or indirectly compromise the spinal nerve and its roots by causing ischemia or inflammation of the axons. The causes are multifactorial. Furthermore, the radicular pain can be evoked by radiculitis caused by viral infection or post-viral inflammation of a dorsal root ganglion, e.g., herpes zoster and postherpetic neuralgia. Arteritis can be also the cause of radiculitis [23]. Pain perception is very subjective and individual. The objective quantification of pain is demanding, multiple questionnaires or surveys are used [24]. The Visual Analogue Scale (VAS) can be used as a simple, valid, and effective way to assess disease control [25,26].

The occurrence of neurological symptoms in COVID-19 can be elucidated also by the neurotropism of the human coronaviruses. Their affinity to the nerve structures was proved and published by Desforges [27], Netland [28], and others [16,29,30,31,32,33]. The infection of nerve structures leads to oedema of the nerves and affects the nerve signal transmission, and further neural structure damage.

In process of neuroinvasion and nervous system damage, various routes are possible, for example trans-synaptic transfer across infected neurons [34], entry via the olfactory nerve [19,20,35], or leukocyte migration across the blood-brain barrier [27,28,31,32,36,37]. Nevertheless, the immunological response might be of high influence on the process of neuroinvasion of coronaviruses in the process of neural symptoms development [37,38,39]. Both direct and indirect mechanisms of nervous tissues impairment may be of relevance. The neurotropism was proven on animal models [28,30,33,38] and in vitro [32,36,40].

Based on these findings we hypothesize that the neurotropism of SARS-CoV-2 can lead to radiculitis. If radiculitis affects the nerve root previously compressed by disc herniation, it leads to worsening of the radicular pain. Verification of this hypothesis was accomplished by the course of worsened radicular pain during acute COVID-19 in nine chronic lumbosacral radicular irritation patients due to lumbosacral disc herniation compromising the nerve root.

## 2. Materials and Methods

Series of cases reports include nine outpatient patients, out of these were three women from 47 to 55 years of age (51.67 ± 4.16 years of age) and six men 41–57 years of age (51.33 ± 5.68 years of age). They all were already indicated for surgical radicular decompression for the lumbosacral radicular pain because of lumbosacral disk herniation at the Department of Neurosurgery, Faculty of Medicine of Slovak Medical University, University Hospital—St. Michal´s Hospital, Bratislava, Slovakia. The disc herniation was verified by Magnetic Resonance Imaging (MRI). While waiting for the surgery, the SARS-CoV-2 infection led to dramatic worsening of lumbosacral radicular pain.

These patients met inclusion criteria for subjects to be allocated to the study. The inclusion criteria included chronic lumbosacral neural root irritation syndrome indicated for neurosurgery operation, worsening of radicular pain in acute COVID-19, SARS-CoV-2 infection proven through polymerase chain reaction (PCR) in the period from 1 January to 31 March 2021, mild or moderate COVID-19 illness [41], and absence of history of even minor back trauma. The exclusion criteria were as follows: no proven SARS-CoV-2 infection or only antigen test positivity, history of vertebral trauma before COVID-19, severe or critical COVID-19, administration to hospital because of COVID-19, need for acute neurosurgical operation. Patients with long COVID-19 symptomatology [42] were excluded too.

The Visual Analogue Scale (VAS) was used for determination of the subjective feel of lumbosacral radicular pain. The patient marked scores from 0 to 10 on a ruler, where the VAS score was ruled by measuring the distance on the 10-cm line between the ‘no pain at all’ and ‘my pain is as bad as it can be’. A higher score indicated greater pain intensity [25]. The VAS was obtained as a standard part of personal examination in an outpatient clinic or by telephone interview at defined time points. We used the prior infection VAS score obtained at the time of neurosurgery procedure indication (1), at disease onset, when the difficulties from neural irritation aggravation led to seek of medical advice (2), and after recovery of COVID-19 (one month after the positive result of SARS-CoV-2 PCR confirmation) (3).

Cohen’s *d* was used to evaluate pre-post infection changes in the VAS score. An effect size of less than 0.2 was considered small, approximately 0.5 was moderate, and greater than 0.8 was large [43]. Effect sizes were calculated with the software program G*power 3.1 (Heinrich-Heine-Universität Düsseldorf, Düsseldorf, Germany) for Mac OS X [44]. Informed consent was acquired from all subjects.

## 3. Results

The nine patients included (six men and three women) were previously monitored as outpatients at the Department of Neurosurgery for lumbosacral disk herniation with direct nerve root compression with lumbosacral radicular pain and were indicated for neurosurgical surgery for nerve root decompression. The group presented sedentary lifestyle [45], heterogeneity in comorbidities, and in body mass index. While waiting for surgery dates, the novel coronavirus infection was acquired by these patients. Radicular pain worsened during the infection. The VAS values of 5.6 ± 1.1 prior to COVID-19 increased to 8.0 ± 1.3 (*d* = 1.99) during the course of infection, indicating that the infection of SARS-CoV-2 worsened the previous pain intensity. However, the VAS values decreased to 5.8 ± 1.1 after COVID-19 recovery. These data show, that the pain intensity decreased to intensity comparable with intensity prior to infection after recovery (Table 1).

The course of COVID-19 was mild to moderate, no hospitalization due to severe COVID-19 was needed in this group. No acute neurosurgical intervention was needed during the duration of the study for lumbosacral radiculopathy deterioration.

## 4. Discussion

As shown, the group of patients with chronic lumbosacral radicular pain because of direct nerve root compression by disc herniation was identified, in whom the SARS-CoV-2 infection caused aggravation and worsening of the neural roots irritation symptomatology. The radicular pain intensity during COVID-19 was found to be higher (VAS: mean, 8.0; SD, 1.322; median, 8.0) compared with pre-infection (mean, 5.56; SD, 1.130; median, 6.0) and led to seeking neurosurgical advice. When the disease was overcome, the radicular pain intensity returned in the majority of cases to pre-infection levels (VAS: mean, 5.78; SD, 1.093; median, 6.0). The calculated Cohen´s *d* proved the large effect size (*d* = 1.99). The radicular pain deterioration had an acute course and dropped to pre-infectious levels spontaneously. There was no need for acute neurosurgical intervention in this group.

Based on our clinical experience and data available we hypothesize that the radicular pain worsening might be remarkably caused by the affection of previously compressed neural roots structures during SARS-CoV-2 infection. The direct viral infection and/or post-viral inflammation of a dorsal root ganglion, accompanied with oedema and neural transmission changes is considered for the worsened radicular pain.

This might be corroborated by experiments performed on animal models [28,30,33,38] or in vitro [32,36,40]. The neural structures involvement in COVID-19 was extensively confirmed in clinical praxis [4,13,16,17,46,47]. Inflammation, oedema, and axonal damage have been shown in autopsies of olfactory bulbs of patients who died due to COVID-19 [20] and the presence of viral particles were proved in cerebrospinal fluid within brain inflammation [29]. The direct and indirect effect of the virus on neural structures together with host immune system activation may lead to neural roots impairment in the structure and function, which leads to neurological deterioration [14,40] and aggravated neurological symptomatology.

Although the detailed mechanism of vertebral pain origin in human coronavirus infections is not known, multiple geneses are suspected [18,27,48]. Nevertheless, the possible affection of direct and indirect infection and immune system effects on not only neuro-radicular, but also musculoskeletal and disco-ligamental structures, and can affect the back and lumbosacral radicular pain genesis process too. This complex activation can accelerate the development or aggravate chronic neurological diseases [27,40] and can lead to long-term sequelae [5,14]. Pain accompanies COVID-19 more than anosmia and ageusia: pain, 69.3%; taste/smell loss, 43.5% [18] or 33.9% of COVD-19 cases [19]. Musculoskeletal pain showed a significant increase in the course of COVID-19 in the back and spine; it was reported in 50.7% of cases [18].

In majority of cases the vertebral pain intensity dropped to pre-infectious levels after disease was overcome. In SARS-CoV-2 infection the pain can surpass other symptoms or can be the only symptom in oligosymptomatic COVID-19 and alone might be the main reason for seeking medical advice [13,18,49]. Nevertheless, back pain can become chronic. Rarely, the pain and radiculopathy in lumbosacral region can be resistant to conservatory therapy and the neurosurgical decompression of nerve roots is necessary [14].

Limitations of the study are as follows: The borders between musculoskeletal and neuropathic pain can be complicated to distinguish in clinical examination. We selected patients with radicular pain, which is strictly a pain problem of the affected limb. The sample size, by reason of this precise selection, is small. The microbial evidence of SARS-CoV-2 in neural roots was not performed, as a biopsy can lead to irreversible damage to the neural root and patients with acute COVID-19 were not indicated for acute neurosurgical intervention. Ongoing COVID-19 the CT/MRI scans were not performed in the acute session. The pain perception is very subjective and individual, and therefore VAS values are under subjective bias. The objective quantification of pain is difficult, and the objective measurement is impossible. The VAS score is easy to use with routine examination.

Studies investigating neurological associations of COVID-19 in vitro and in vivo are necessary to increase our knowledge base. On the other hand, as new mutations of SARS-CoV-2 [50,51] with possibly changed biological characteristics occur, the prospective comparable clinical trials will be difficult to design and evaluate. With the omicron variant pathogenicity [52], the hope that possible new variants of SARS-CoV-2 are less severe and have only minor impacts on human health is rising.

## 5. Conclusions

The worsened radicular pain accompanies the SARS-CoV-2 infection in patients who suffer from chronic lumbosacral radicular irritation resulting from previous nerve roots compression by intervertebral disc herniation. In the majority of cases the radicular pain intensity decreased spontaneously after COVID-19 recovery to pre-infectious levels. The acute aggravation and deterioration of chronic difficulties can be considered the prominent symptom of the COVID-19, which may be ascribed to SARS-CoV-2 radiculitis of neural roots compressed by the previous disc herniation. These findings based on clinical observations and literature sources indicate that the neurotropism of the novel coronavirus can perform an important role in the neural root irritation symptomatology deterioration in patients with chronic pre-existing lumbosacral radicular syndrome.

## Figures and Tables

**Table 1:** Radicular pain intensity changes during the course of COVID-19.

Patient	Sex	Age (Years)	Pre-COVID-19 VAS	Peri-COVID-19 VAS	Post-COVID-19 VAS
1	M	41	7	10	7
2	F	47	6	9	7
3	M	51	4	8	5
4	M	50	7	9	7
5	F	53	6	7	6
6	M	54	4	6	4
7	F	55	6	9	6
8	M	55	5	7	5
9	M	57	5	7	5

M: male; F: female; VAS: Visual Analogue Scale.

## Data Availability

The data presented in this study are available in the article.

## References

[1] COVID-19 Dashboard. https://coronavirus.jhu.edu/map.html.

[2] Chen X., Zheng F. Epidemiological and Clinical Features of 291 Cases with Coronavirus Disease 2019 in Areas Adjacent to Hubei, China: A Double-Center Observational Study. https://www.medrxiv.org/content/10.1101/2020.03.03.20030353v1.

[3] Guan W.J., Ni Z.Y. (2020). China Medical Treatment Expert Group for COVID-19. Clinical characteristics of coronavirus disease 2019 in China. N. Engl. J. Med..

[4] Raveendran A.V., Jayadevan R., Sashidharan S. (2021). Long COVID: An overview. Diabetes Metab. Syndr..

[5] Lopez-Leon S., Wegman-Ostrosky T., Perelman C. (2021). More than 50 Long-term effects of COVID-19: A systematic review and meta-analysis. Sci. Rep..

[6] WHO China Joint Mission on COVID-19 Final Report. https://www.who.int/docs/default-source/coronaviruse/who-china-joint-mission-on-covid-19-final-report.pdf.

[7] Paliwal V.K., Garg R.K., Gupta A., Tejan N. (2020). Neuromuscular presentations in patients with COVID-19. Neurol. Sci..

[8] Bartoš H., Fabianová L., Dlouhý P.D. (2021). Ospalík.: COVID-19 asociovaná myelitida—Kazuistika vzácné komplikace závažné SARS-CoV-2 infekce (COVID-19 associated myelitis—A case report of rare complication of severe SARS-CoV-2 infection). Ces. Slov. Neurol. Neurochir..

[9] Munz M., Wessendorf S., Koretsis G., Tewald F., Baegi R., Krämer S., Geissler M., Reinhard M. (2020). Acute transverse myelitis after COVID-19 pneumonia. J. Neurol..

[10] AlKetbi R., AlNuaimi D., AlMulla M., AlTalai N., Samir M., Kumar N., AlBastaki U. (2020). Acute myelitis as a neurological complication of Covid-19: A case report and MRI findings. Radiol. Case Rep..

[11] Zhao K., Huang J., Dai D., Feng Y., Liu L., Nie S. (2020). Acute myelitis after SARS-CoV-2 infection: A case report (PREPRINT). MedRxiv.

[12] Guo F., Zhang Y.B. (2019). Clinical features and prognosis of patients with Guillain-Barré and acute transverse myelitis overlap syndrome. Clin. Neurol. Neurosurg..

[13] Chochol J., Džubera A., Illéš R., Chocholová A., Zemková E. (2021). Vertebral Pain in Acute COVID-19—Cases Report. Appl. Sci..

[14] Džubera A., Chochol J., Illéš R., Chocholová A., Zemková E. (2021). Vertebral Algic Syndrome Treatment in Long COVID-Cases Reports. Int. J. Environ. Res. Public Health.

[15] Niazkar H.R., Zibaee B., Nasimi A. (2020). The neurological manifestations of COVID-19: A review article. Neurol. Sci..

[16] Yachou Y., El Idrissi A., Belapasov V., Ait Benali S. (2020). Neuroinvasion, neurotropic, and neuroinflammatory events of SARS-CoV-2: Understanding the neurological manifestations in COVID-19 patients. Neurol. Sci..

[17] Mao L., Jin H., Wang M., Hu Y., Chen S., He Q., Chang J., Hong C., Zhou Y., Wang D. (2020). Neurologic manifestations of hospitalized patients with coronavirus disease 2019 in Wuhan, China. JAMA Neurol..

[18] Murat S., Karatekin B.D. (2021). Clinical presentations of pain in patients with COVID-19 infection. Ir. J. Med. Sci..

[19] Giacomelli A., Pezzati L. (2020). Self-reported Olfactory and Taste Disorders in Patients with Severe Acute Respiratory Coronavirus 2 Infection: A Cross-sectional Study. Clin. Infect. Dis..

[20] Kirschenmbaum D., Imbach L.L., Ulrich S., Rushing E.J., Keller E., Reimann R.R., Frauenknecht K.B., Lichtblau M., Witt M., Hummel T. (2020). Inflammatory olfactory neuropathy in two patients with Covid-19. Lancet.

[21] Paybast S., Gorji R., Mavandadi S. (2020). Guillain-Barré syndrome as a neurological complication of novel COVID-19 infection: A case report and review of the literature. Neurologist.

[22] Moreira M.S., Neves I.L.I., de Bernoche C.Y.S.M., Sarra G., dos Santos-Paul M.A., da Silva F.C.N., Schroter G.T., Montano T.C.P., de Carvalho C.M.A., Neves R.S. (2022). Bilateral paresthesia associated with cardiovascular disease and COVID-19. Oral Dis..

[23] Merskey H., Bogduk N. (2012). Classification of Chronic Pain. www.iasp-pain.org/publications/free-ebooks/classification-of-chronic-pain-second-edition-revised/.

[24] Karcioglu O., Topacoglu H., Dikme O., Dikme O. (2018). A systematic review of the pain scales in adults: Which to use?. Am. J. Emerg. Med..

[25] Crichton N. (2001). Visual analogue scale (VAS). J. Clin. Nurs..

[26] Klimek L., Bergmann K.C., Biedermann T., Bousquet J., Hellings P., Jung K., Merk H., Olze H., Schlenter W., Stock P. (2017). Visual analogue scales (VAS): Measuring instruments for the documentation of symptoms and therapy monitoring in cases of allergic rhinitis in everyday health care. Allergo J. Int..

[27] Desforges M., Le Coupanec A., Stodola J.K., Meessen-Pinard M., Talbot P.J. (2014). Human coronaviruses: Viral and cellular factors involved in neuroinvasiveness and neuropathogenesis. Virus Res..

[28] Netland J., Meyerholz D.K., Moore S., Cassell M., Perlman S. (2008). Severe acute respiratory syndrome coronavirus infection causes neuronal death in the absence of encephalitis in mice transgenic for human ACE2. J. Virol..

[29] Yeh E.A., Collins A., Cohen M.E., Duffner P.K., Faden H. (2004). Detection of coronavirus in the central nervous system of a child with acute disseminated encephalomyelitis. Pediatrics.

[30] Natoli S., Oliveira V., Calabresi P., Maia L.F., Pisani A. (2020). Does SARS-Cov-2 invade the brain? Translational lessons from animal models. Eur. J. Neurol..

[31] Gu J., Gong E., Zhang B., Zheng J., Gao Z., Zhong Y., Zou W., Zhan J., Wang S., Xie Z. (2005). Multiple organ infection and the pathogenesis of SARS. J. Exp. Med..

[32] Spiegel M. (2006). Interaction of severe acute respiratory syndrome-associated coronavirus with dendritic cells. J. Gen. Virol..

[33] Hao X., Lv Q., Li F., Xu Y., Gao H. (2019). The characteristics of hDPP4 transgenic mice subjected to aerosol MERS coronavirus infection via an animal nose-only exposure device. Anim. Models Exp. Med..

[34] Conde Cardona G., Quintana Pájaro L.D., Quintero Marzola I.D., Ramos Villegas Y., Moscote Salazar L.R. (2020). Neurotropism of SARS-CoV 2: Mechanisms and manifestations. J. Neurol. Sci..

[35] Brann D.H., Tsukahara T., Weinreb C., Logan D.W., Datta S.R. (2020). Non-neural expression of SARS-CoV-2 entry genes in the olfactory system suggests mechanisms underlying COVID-19-associated anosmia. Sci. Adv..

[36] Bayati A., Kumar R., Francis V., McPherson P.S. (2021). SARS-CoV-2 infects cells after viral entry via clathrin-mediated endocytosis. J. Biol. Chem..

[37] Zhou J., Chu H., Li C., Wong B.H.Y., Cheng Z.S., Poon V.K.M., Sun T., Lau C.C.Y., Wong K.K.Y., Chan J.Y.W. (2014). Active replication of Middle East respiratory syndrome coronavirus and aberrant induction of inflammatory cytokines and chemokines in human macrophages: Implications for pathogenesis. J. Infect. Dis..

[38] Li Y.C., Bai W.Z., Hirano N., Hayashida T., Hashikawa T. (2012). Coronavirus infection of rat dorsal root ganglia: Ultrastructural characterization of viral replication, transfer, and the early response of satellite cells. Virus Res..

[39] Li Y., Fu L., Gonzales D.M., Lavi E. (2004). Coronavirus neurovirulence correlates with the ability of the virus to induce proinflammatory cytokine signals from astrocytes and microglia. J. Virol..

[40] Arbour N., Day R., Newcombe J., Talbot P.J. (2000). Neuroinvasion by human respiratory coronaviruses. J. Virol..

[41] COVID-19 Treatment Guidelines. https://www.covid19treatmentguidelines.nih.gov/overview/clinical-spectrum/.

[42] Greenhalgh T., Knight M., Court C. (2020). Management of post-acute covid-19 in primary care. BMJ.

[43] Cohen J. (1988). Statistical Power Analysis for the Behavioral Science.

[44] Faul F., Erdfelder E. (2007). G*Power 3: A flexible statistical power analysis program for the social, behavioral, and biomedical sciences. Behav. Res. Methods.

[45] Booth F.W., Lees S.J. (2006). Physically active subjects should be the control group. Med. Sci. Sports Exerc..

[46] Zubair A.S., McAlpine L.S., Gardin T., Farhadian S., Kuruvilla D.E., Spudich S. (2020). Neuropathogenesis and Neurologic Manifestations of the Coronaviruses in the Age of Coronavirus Disease 2019: A Review. JAMA Neurol..

[47] Gialluisi A., de Gaetano G., Iacoviello L. (2020). New challenges from Covid-19 pandemic: An unexpected opportunity to enlighten the link between viral infections and brain disorders?. Neurol. Sci..

[48] Sarkesh A., Daei Sorkhabi A. (2020). Extrapulmonary Clinical Manifestations in COVID-19 Patients. Am. J. Trop. Med. Hyg..

[49] Cipollaro L., Giordano L. (2020). Musculoskeletal symptoms in SARS-CoV-2 (COVID-19) patients. J. Orthop. Surg. Res..

[50] Classification of Omicron (B.1.1.529): SARS-CoV-2 Variant of Concern. https://www.who.int/news/item/26-11-2021-classification-of-omicron-(b.1.1.529)-sars-cov-2-variant-of-concern.

[51] SARS-CoV-2 Variant Classifications and Definitions. https://www.cdc.gov/coronavirus/2019-ncov/variants/variant-classifications.html.

[52] Chen J., Wang R., Gilby N.B., Wei G.W. (2022). Omicron Variant (B.1.1.529): Infectivity, Vaccine Breakthrough, and Antibody Resistance. J. Chem. Inf. Model..

